# Supplementary material for: Extrafollicular CD4+ T-B interactions are sufficient for inducing autoimmune-like chronic graft-versus-host disease
Source: Nat Commun. 2017 Oct 17;8:978. doi: 10.1038/s41467-017-00880-2 (PMC5645449; doi:10.1038/s41467-017-00880-2)
Supplement: Supplementary file 1 — Supplementary Information [file 41467_2017_880_MOESM1_ESM.pdf]

Supplementary figures:

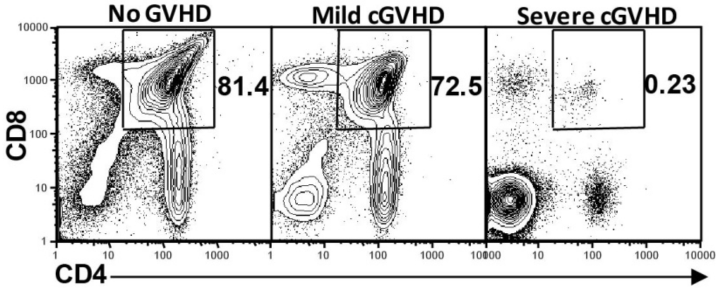

Supplementary Fig. 1 Representative flow patterns of thymocytes in recipients with different severity of cGVHD. BALB/c mice were irradiated (850 cGy) and given $2.5x10^6$ TCD-BM (T cell depleted bone marrow) alone (n=4) or $2.5x10^6$ TCD-BM plus $1X10^6$ (n=4) or $0.01x10^6$ (n=4) splenocytes from C57BL/6 donors. 60 days after transplantation thymus specimens were harvested and stained with CD4 and CD8. Gated mononuclear cells are shown as CD4 versus CD8. One representative of 4 recipients in each group is shown.

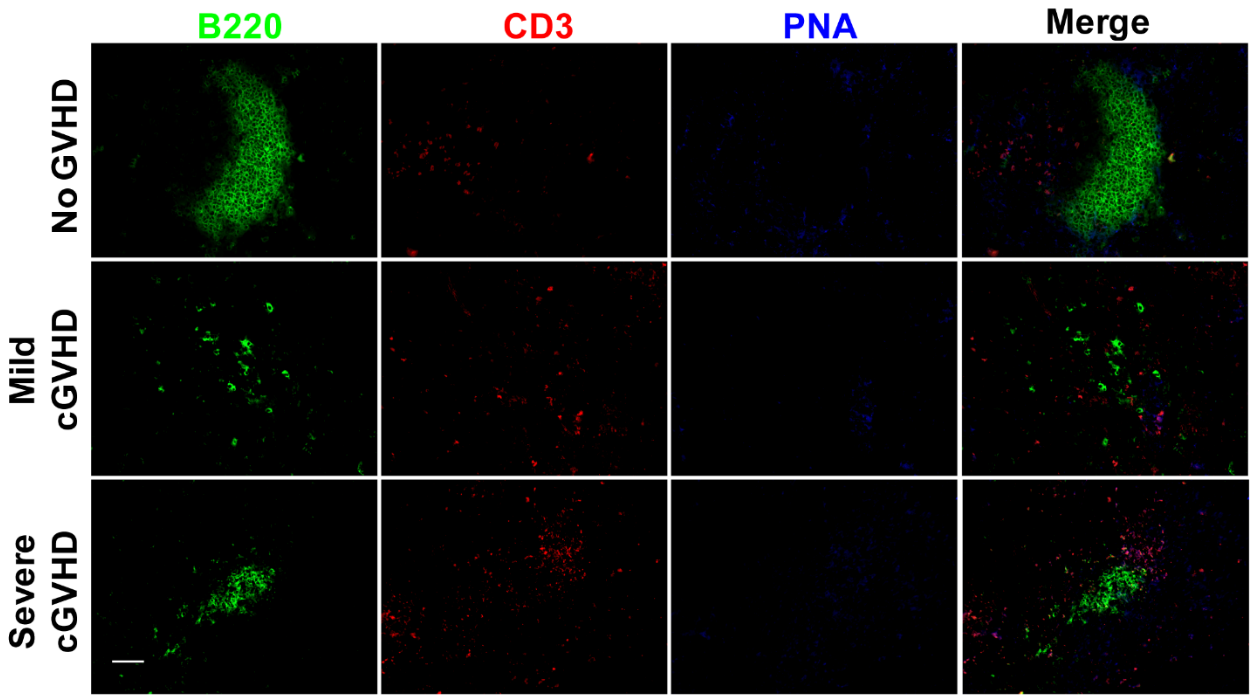

Supplementary Fig. 2 No germinal centers were observed in BALB/c recipients on day 15 after HCT. BALB/c mice were irradiated (850 cGy) and given 2.5×10^6 TCD-BM alone or 2.5×10^6 TCD-BM plus $1 \mathrm{X} 10^6$ or 0.01×10^6 (n=6) splenocytes from C57BL/6 donors. Immunofluorescent staining pattern of B220, CD3 and PNA on cryosections of spleen harvested at 15 days after transplantation. One representative of 6 recipients in each group is shown. Scale bar,50μm.

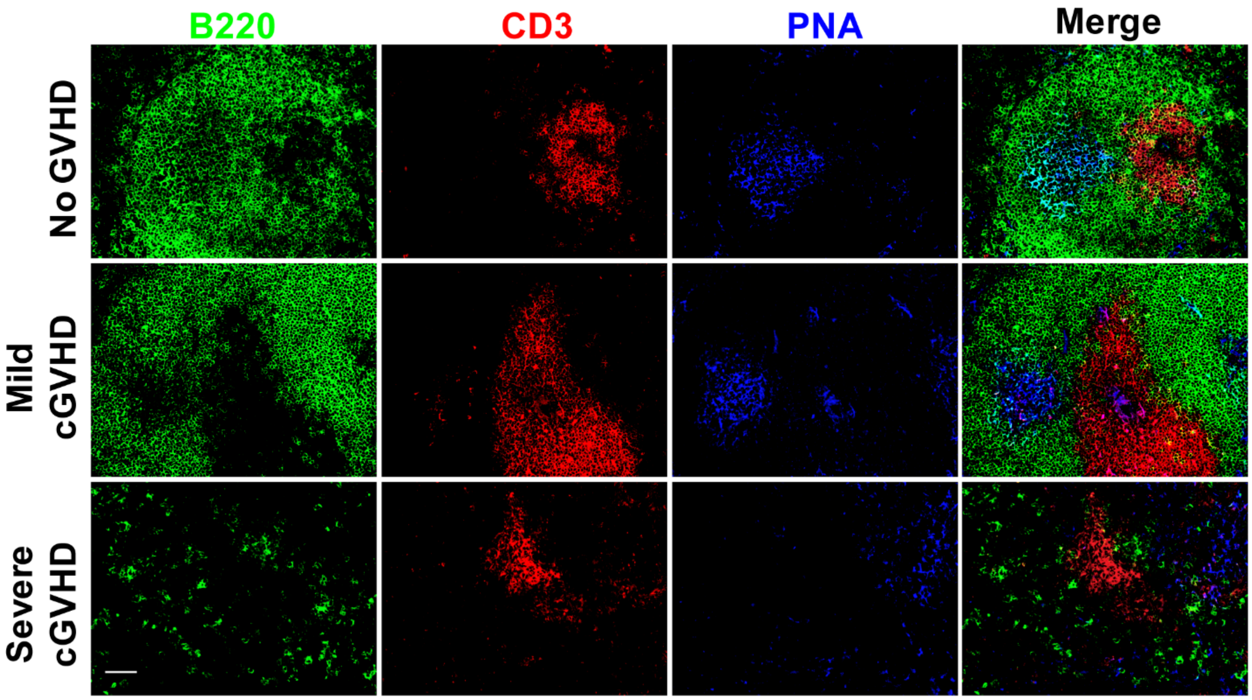

Supplementary Fig. 3 Germinal center formation was not observed in BALB/c recipients with severe GVHD. BALB/c mice were irradiated (850 cGy) and given $2.5x10^6$ TCD-BM alone or $2.5x10^6$ TCD-BM plus $1X10^6$ or $0.01x10^6$ (n=6) splenocytes from C57BL/6 donors. Immunofluorescent staining of B220, CD3 and PNA on cryosections of spleen harvested at 30 days after transplantation. One representative of 6 recipients in each group is shown. Scale bar,50µm.

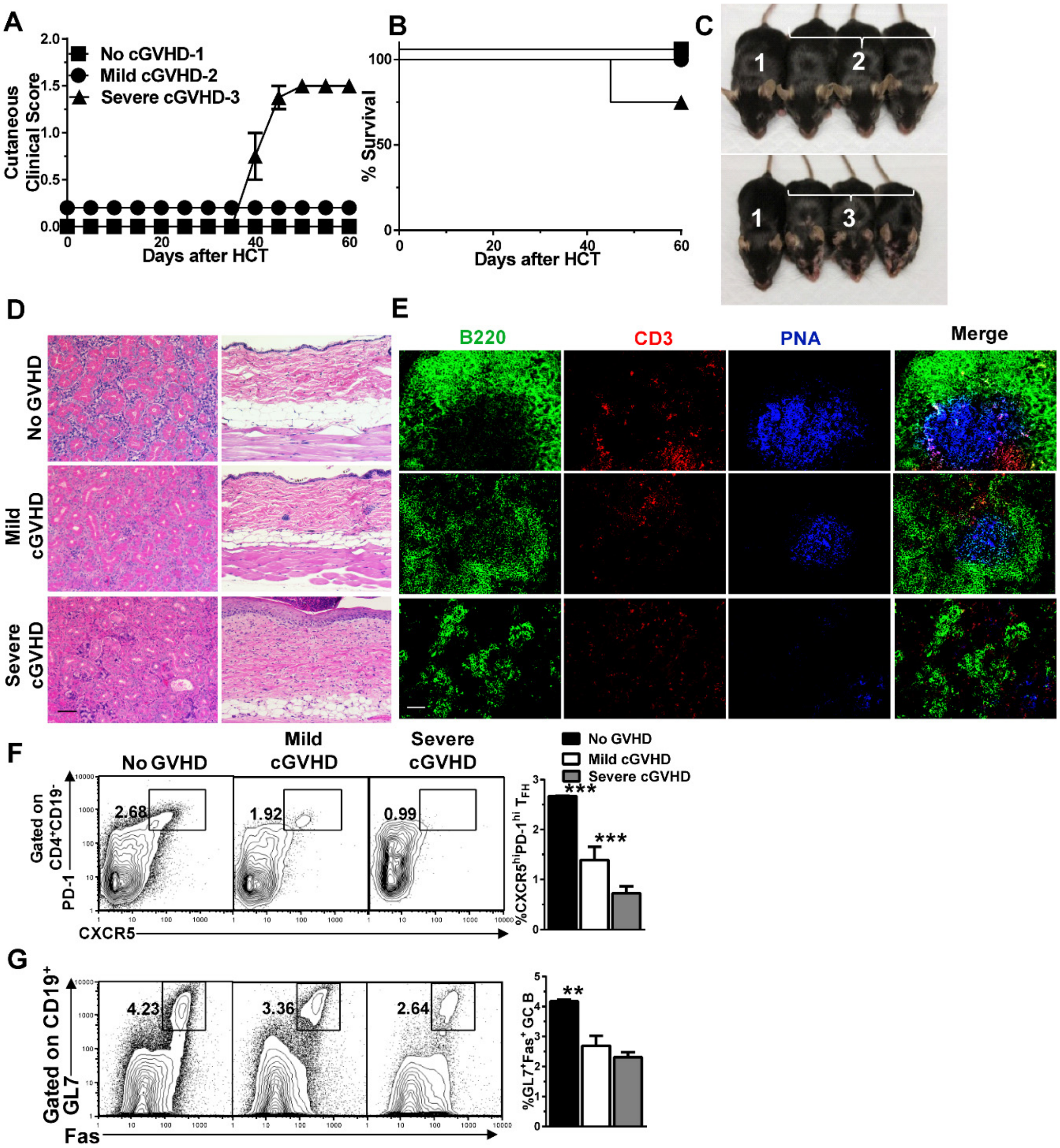

Supplementary Fig. 4 No germinal center formation in chronic GVHD C57BL/6 recipients given LP/J grafts. Lethally irradiated C57BL/6 recipients were given whole spleen (10×10^6) and TCD-BM (2.5×10^6) from LP/J donors. Recipients were monitored for cGVHD for up to 60 days (N=12). (A) Cutaneous GVHD scores (Group 3 vs Group 1: P<0.001, Group 3 vs Group 2: P<0.001, two-way ANOVA test). (B)Survival curve. (C) A representative photograph of a GVHD-free recipient, 3 recipients with mild GVHD, and 3 recipients with facial hair-loss at 60 days after HCT. (D) One representative of 8 skin and salivary gland histopathology evaluation is shown. Scale bar, 50μm (E) 60 days afer HCT, splenic cryosections were analyzed by immunofluorescent staining for lymphoid follicle structure and GC formation. B220 (Green) and CD3 (red) were used to show the B and T cell zone of lymphoid follicle, respectively. Peanut Agglutinin (PNA, blue) was used to detect GC B cells. One representative of 4 recipients in each group is shown. Scale bar, 50μm (F) Splenocytes were stained for CD4, CD19, CXCR5, and PD-1. Gated donor $CD4^+CD19^-$ cells are shown as CXCR5 versus PD-1. $CXCR5^{hi}PD\text{-}1^{hi}$ cells were gated as T_{FH}. Percentages of $CXCR5^{hi}PD\text{-}1^{hi}$ cells among $CD4^+CD19^-$ cells are shown as mean ± SE (N=4). (G) Splenocytes were stained with CD19, Fas and GL7. Gated $CD19^+$ cells are shown as GL7 versus Fas. $GL7^+Fas^+$ cells were gated as germinal center B cells. Percentages of $GL7^+Fas^+$among $CD19^+$ cells are shown as mean ± SE (N=4). ** P<0.01, ***P<0.001, unpaired 2-tailed student t test.

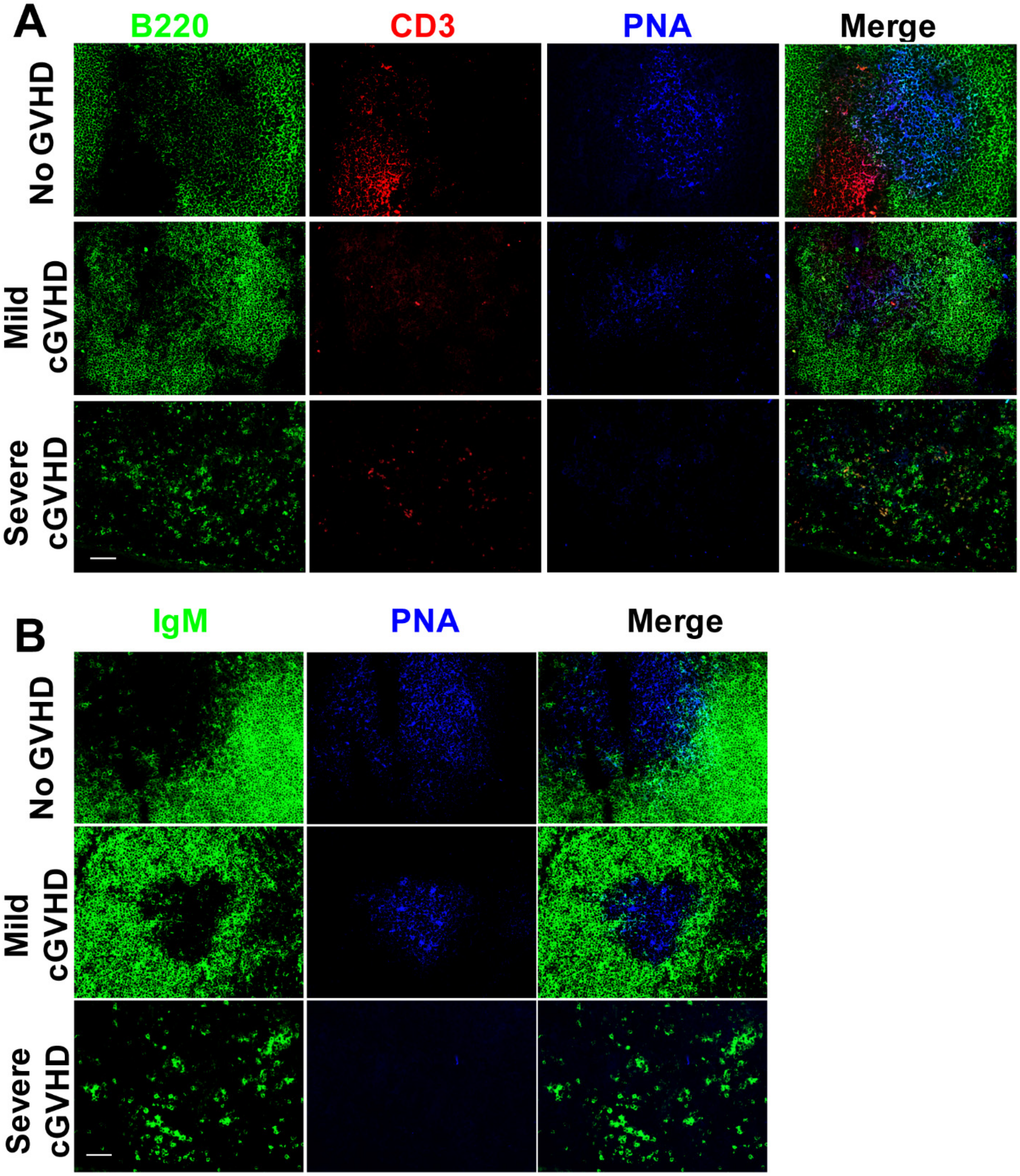

Supplementary Fig. 5 Different combination of staining reagents fail to detect GC formation in B10.BR recipients with severe chronic GVHD. Lethally irradiated B10.BR recipients were given 2.5×10^6 TCD-BM alone or 2.5×10^6 TCD-BM plus 1×10^6 or 0.1×10^6 splenocytes. 28 days after HCT, spleens were harvested. (A) Splenic cryosections were analyzed by immunofluorescent staining of lymphoid follicle structure and GC formation. B220 (Green) and CD3 (red) were used to show the B and T cell zones of lymphoid follicles, respectively. Peanut Agglutinin (PNA, blue) was used to detect GC B cells. (B) IgM (Green) was used to show the B cell zone of lymphoid follicles. Peanut Agglutinin (PNA, blue) was used to detect GC B cells. One representative of 4 recipients in each group is shown. Scale bar, 50μm.

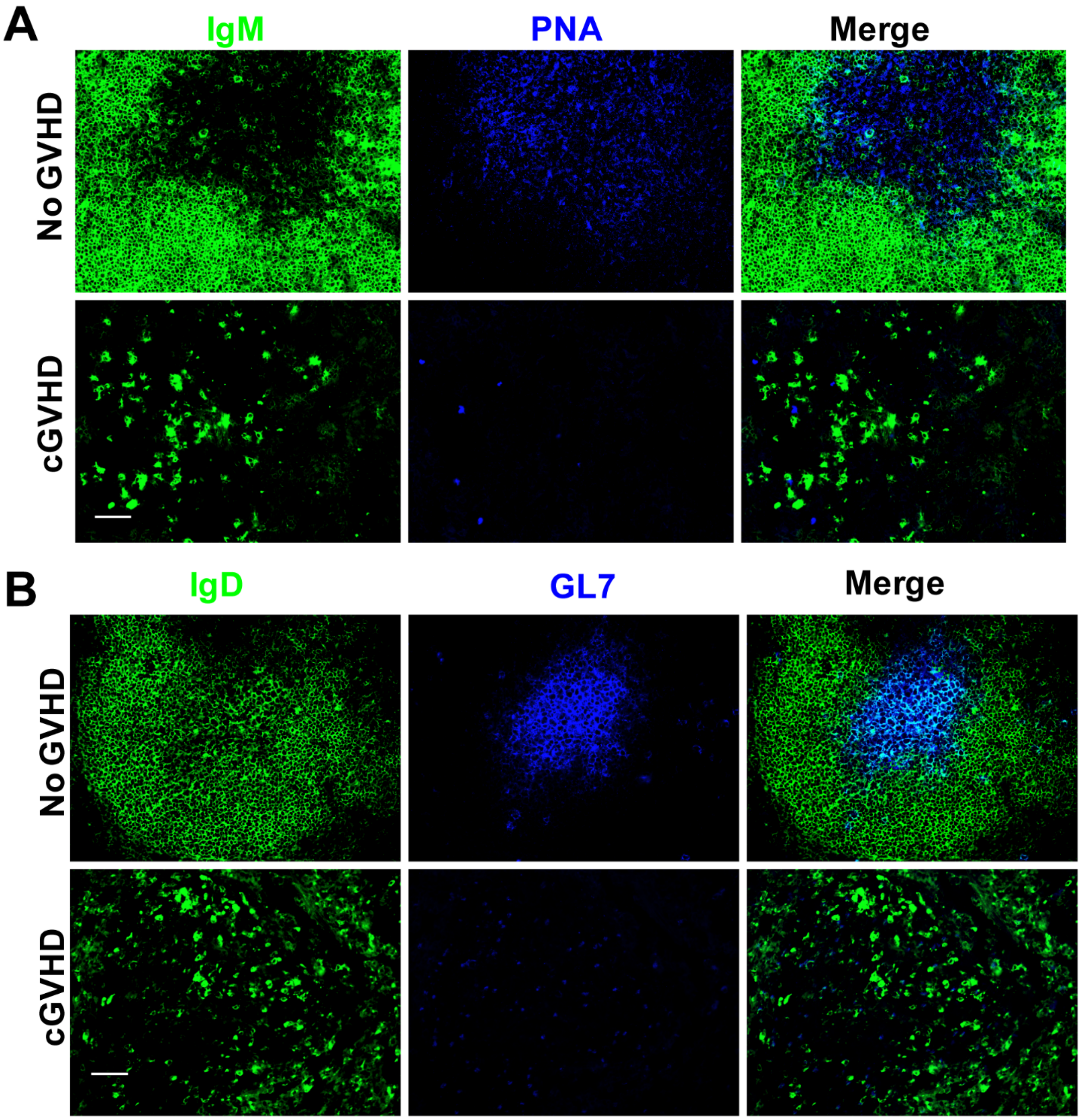

Supplementary Fig. 6 Different combination of staining reagents fail to detect GC formation in C57BL/6 recipients with severe chronic GVHD. Lethally irradiated C57BL/6 mice were given with whole spleen ($10×10^6$) and TCD-BM ($2.5×10^6$) from LP/J donors. 60 days after HCT, splenic sections of recipients were analyzed by immunofluorescent staining for lymphoid follicle structure and GC formation. (A) IgM (Green) was used to show the B cell zone of lymphoid follicle. Peanut Agglutinin (PNA, blue) was used to detect GC B cells. (B) IgD (Green) was used to show the B cell zone of lymphoid follicles. GL7 (blue) was used to detect GC B cells. One representative of 4 recipients in each group is shown. Scale bar, 50μm.

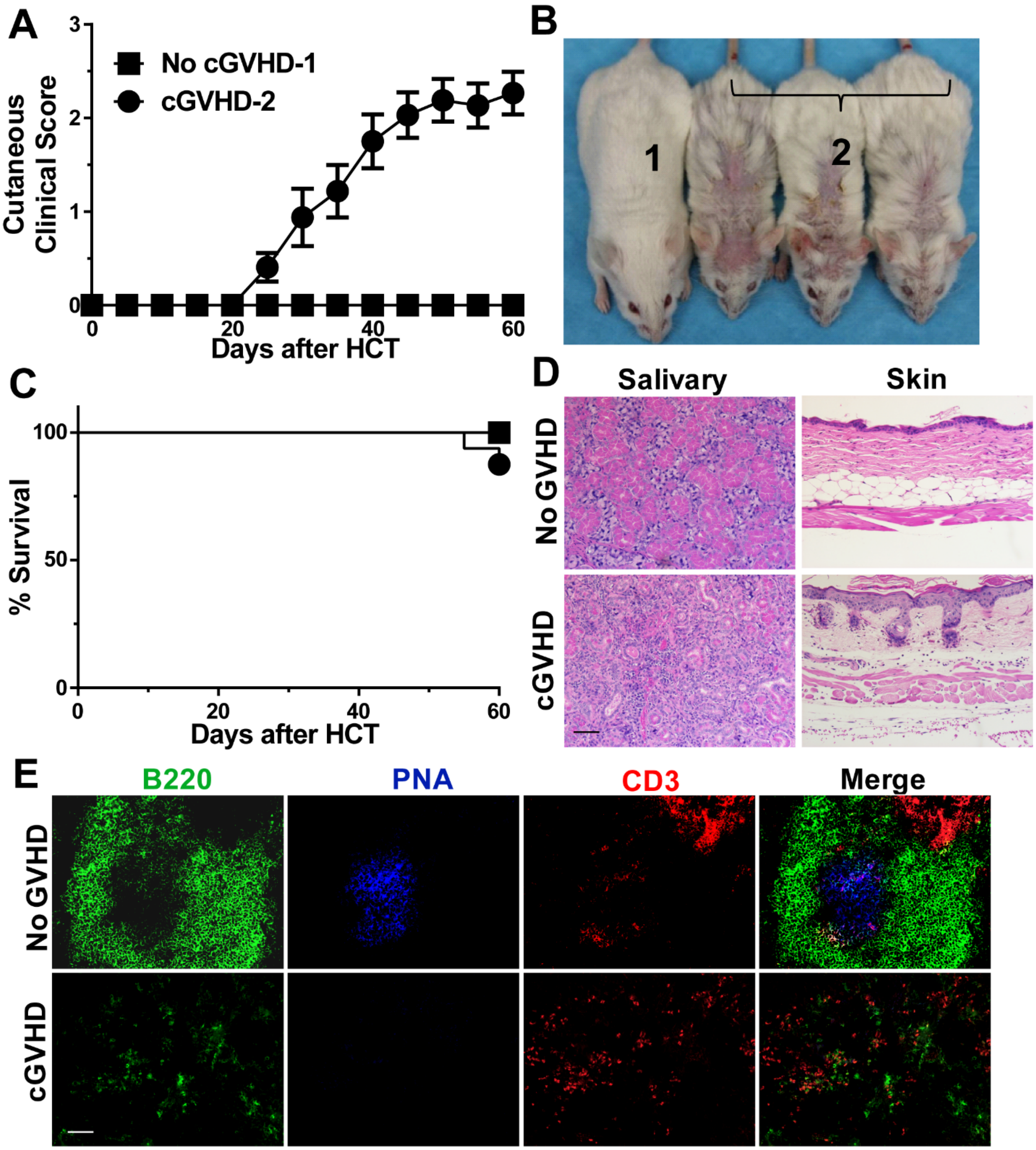

Supplementary Fig. 7 No germinal center formation in BALB/c recipients given MHC-matched B10.D2 grafts. Lethally irradiated BALB/c mice were transplanted with whole spleen (10×10^6) and TCD-BM (2.5×10^6) from B10.D2 donors. The recipients were monitored for cGVHD development for up to 60 days (N=12). (A) Cutaneous GVHD score (Group 2 vs Group 1: P<0.001 two-way ANOVA). (B) A representative photograph of 1 GVHD-free and 3 cGVHD recipients at day 60 after HCT. (C) Survival curve. (D) One representative skin and salivary gland histopathology is shown. (E) B220 (green) and CD3 (red) were used to show the B and T cell zones of lymphoid follicles, respectively. Peanut Agglutinin (PNA, blue) was used to detect GC B cells. One representative of 4 recipients in each group is shown. Scale bars, 50μm.

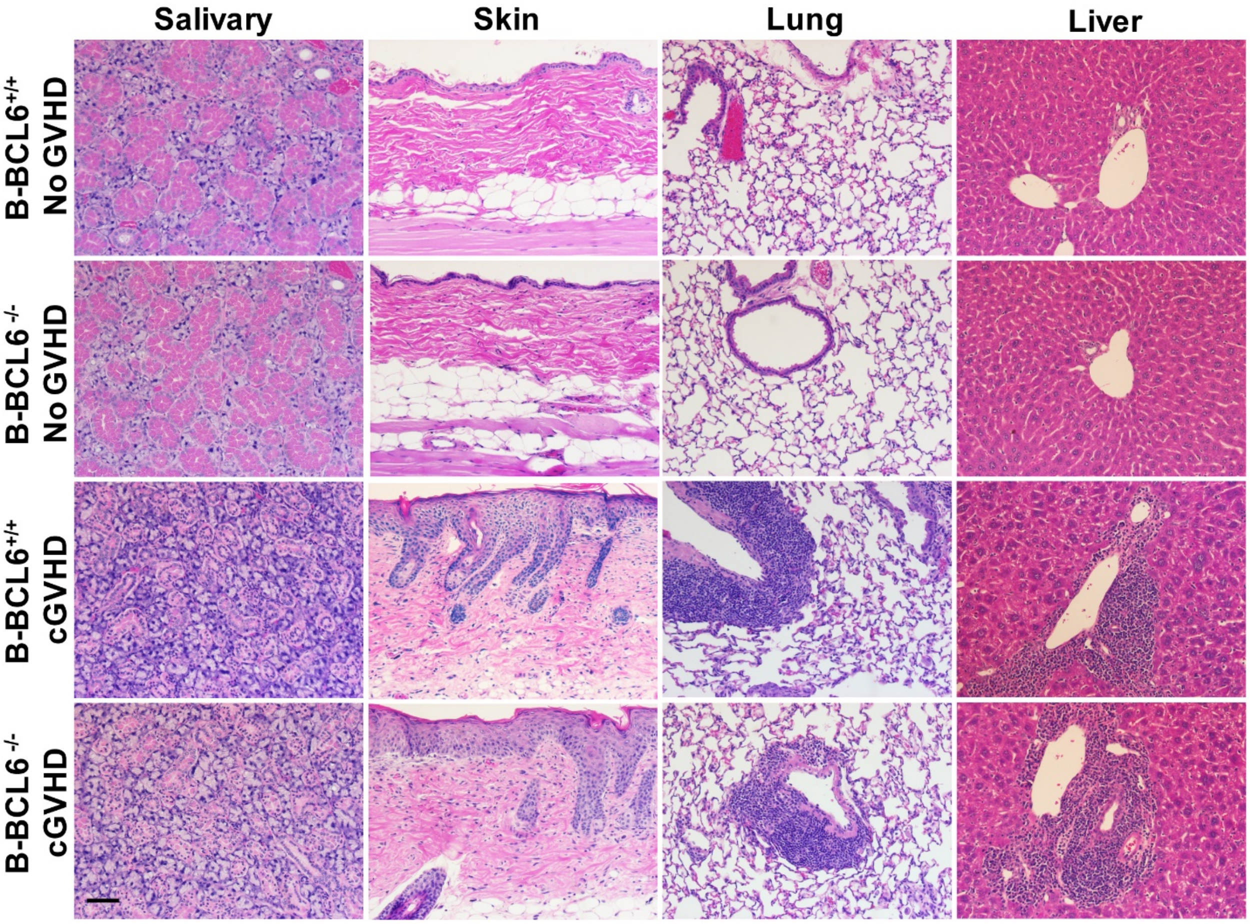

Supplementary Fig. 8 Histopathology of salivary gland, skin, lung and liver of BALB/c recipients given grafts with BCL6$^{-/-}$ B cells. BALB/c recipients were irradiated with 850 cGy and given 2.5x10^{6} TCD-BM alone or 2.5x10^{6} TCD-BM plus 1X10^{6} splenocytes from either WT or B-BCL6$^{-/-}$ C57BL/6 donors. cGVHD was monitored. 60 days after HCT, salivary gland, skin, lung and liver specimens were harvested and used for H&E staining. One representative of 6 recipients in each group is shown. Scale bar, 50μm.

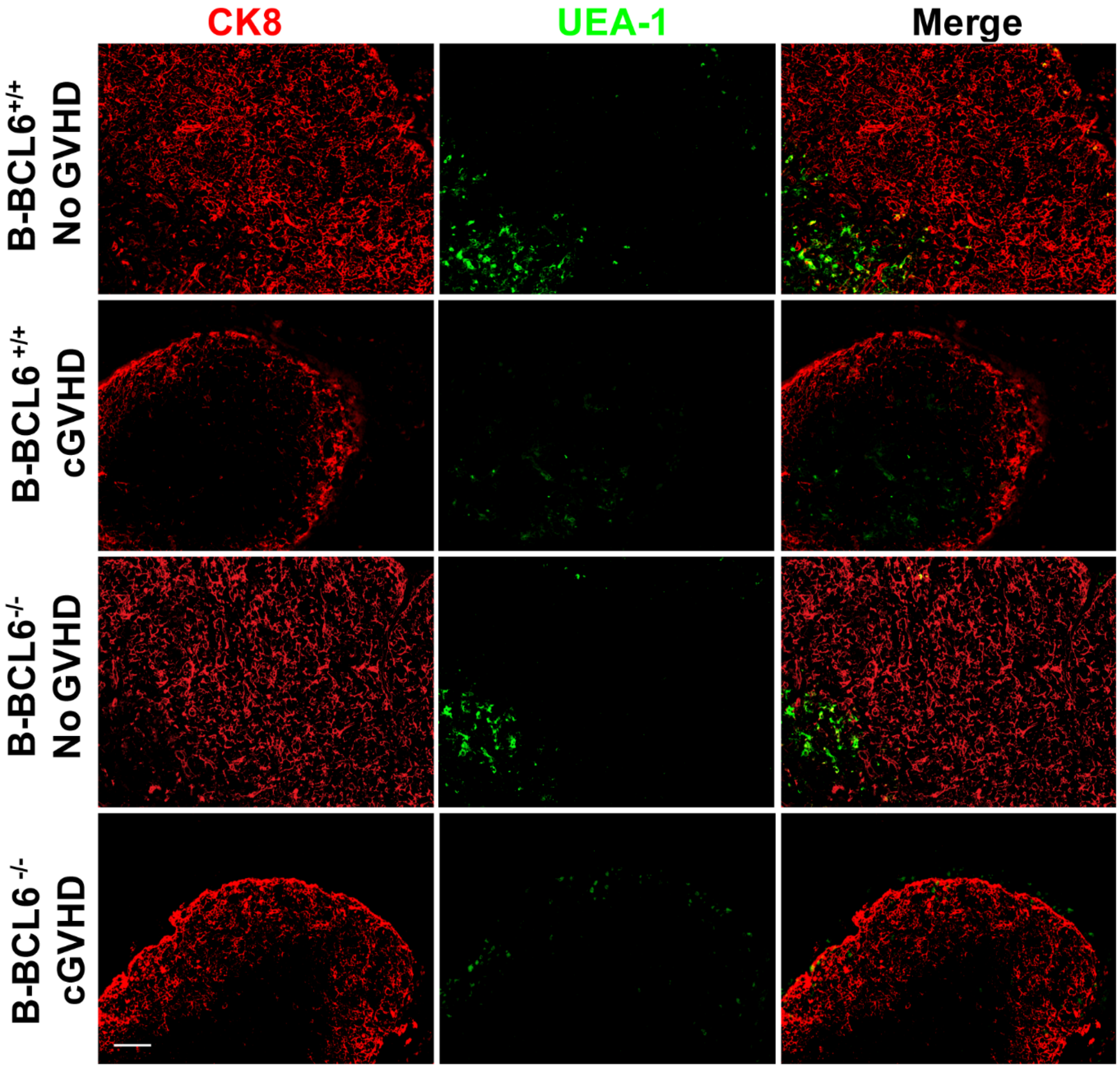

Supplementary Fig. 9 Severe thymus damage in cGVHD recipients given B-BCL6$^{-/-}$ grafts. 60 days after HCT, thymus specimens were harvested and stained with CK8 for cortex epithelial cells and UEA-1 for medulla epithelial cells. One representative of 6 recipients in each group is shown. Scale bar, 50μm.

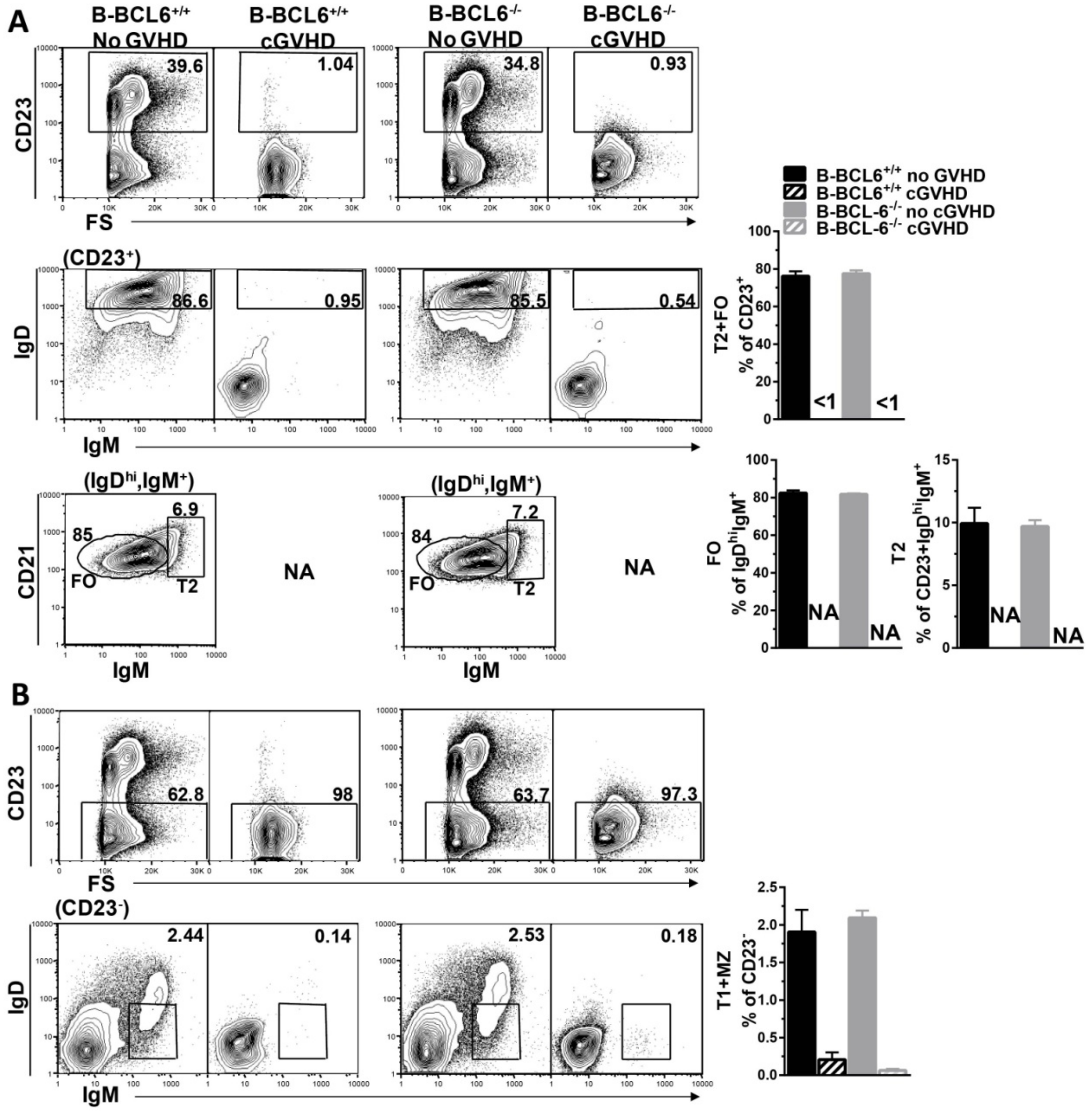

Supplementary Fig. 10 Flow cytometry analysis of follicular B, T2 B, and T1 plus marginal zone B cells in recipients given B-BCL6$^{-/-}$ grafts. (A) 60 days after HCT, splenocytes were stained for CD23, IgM, IgD and CD21. Gated CD23^{+} cell is shown as IgM versus IgD. Gated IgM^{hi}IgD^{hi} cells are shown as IgM versus CD21. Percentages of CD23^{+}, T2B (CD23^{+}IgD^{hi}IgM^{hi}CD21^{+}) cell and follicular B cells (CD23^{+}IgD^{hi}IgM^{lo}CD21^{+}) cells are shown as mean ± SE (n=6) (B) Gated CD23^{-} cells are shown as IgM versus IgD. Percentages of T1+MZ cells (CD23^{-}IgD^{lo}IgM^{hi}) are shown as mean ± SE (n=6).

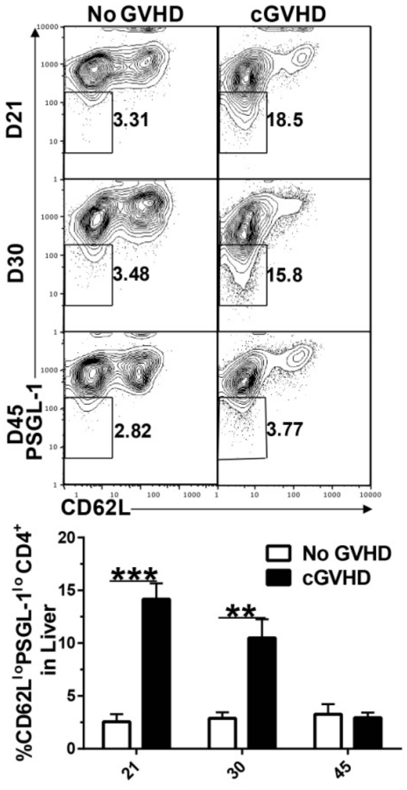

Supplementary Fig. 11 $CD4^{+}CD44^{hi}$ $CD62L^{lo}PSGL\text{-}1^{lo}$ T cell population in the liver of cGVHD BALB/c recipients. BALB/c recipients were irradiated (850 cGy) and given $2.5x10^{6}$ TCD-BM or $2.5x10^{6}$ TCD-BM plus $1x10^{6}$ splenocytes. 21, 30, 45 days after HCT, mononuclear cells isolated from liver were stained for CD4, CD44, PSGL-1 and CD62L. Gated $CD4^{+}CD44^{hi}$ are shown as PSGL-1 versus CD62L. Percentage of $PSGL\text{-}1^{lo}CD62L^{lo}$ cells among $CD4^{+}CD44^{hi}$ cells are shown as mean ± SE (n=8). **$p<0.01$, *** $p<0.001$ unpaired 2-tailed student t test.

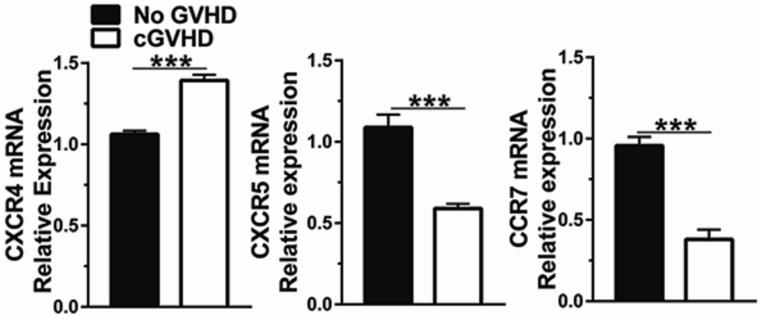

Supplementary Fig. 12 CXCR4 CXCR5 and CCR7 mRNA level tested by real time PCR. 21 days after HCT, splenocytes from no GVHD or chronic GVHD recipients given wild-type C57BL/6 donors were harvested and stained for CD4, CD44, PSGL-1 and CD62L. $CD44^{hi}CD62L^{lo}PSGL\text{-}1^{lo}CD4^{+}$ T cells were sorted and used for RNA isolation. mRNA level of CXCR4, CXCR5 and CCR7 was measured by Real time PCR. Mean ± SE is shown of 4 replicate experiments. Each experiment is combined from 8 mice, *** p<0.001 unpaired 2-tailed student t test.

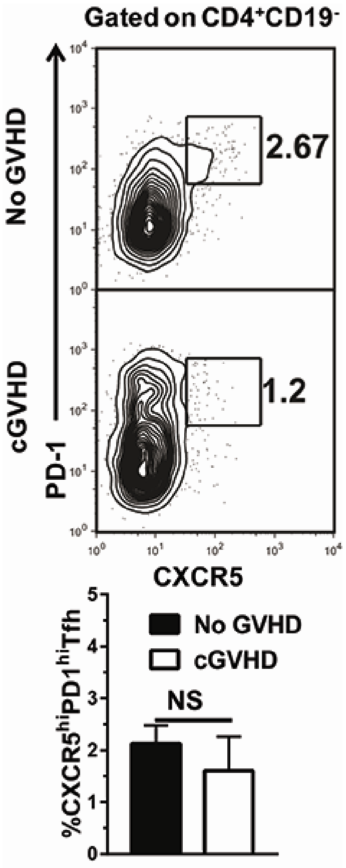

Supplementary Fig. 13 No Expansion of Tfh was observed in BALB/c recipients given MHC-mismatched C57BL/6 grafts 21 days after HCT. Lethally irradiated BALB/c recipients were given whole spleen (1×10^6) and TCD-BM (2.5×10^6) from C57BL/6 donors. 21 days after HCT splenocytes were stained for CD4, CD19, CXCR5 and PD-1. Tfh were gated as $CD4^+CD19^-$ and are shown as $CXCR5^{hi}PD\text{-}1^{hi}$. Percentages of $CXCR5^{hi}PD\text{-}1^{hi}$ cells among $CD4^+CD19^-$ cells were shown as mean ± SE (n=6). NS, unpaired 2-tailed student t test.

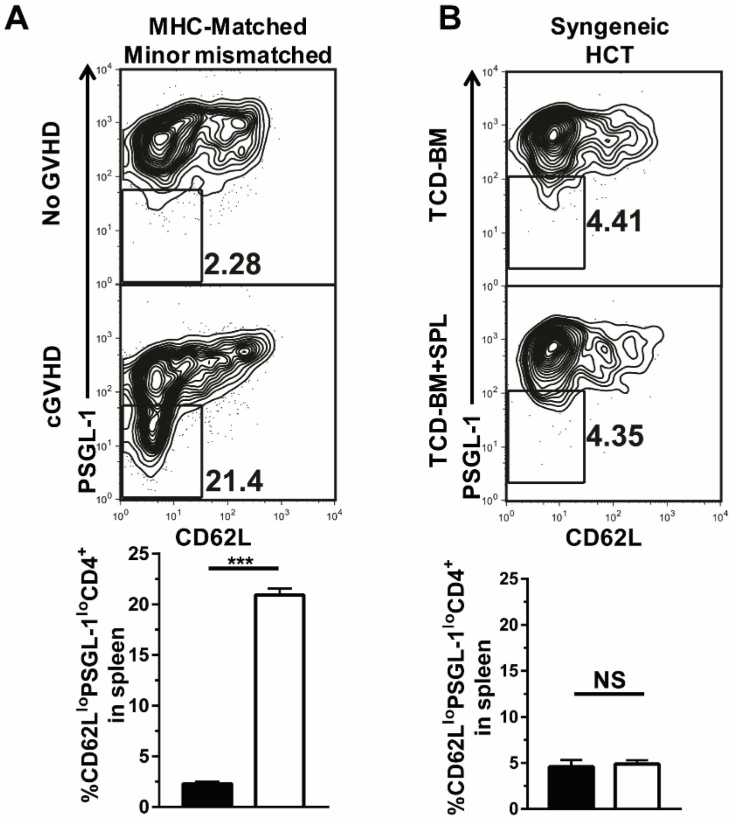

Supplementary Fig. 14 Expansion of PSGL-1^{lo}CD4^{+} T cells in cGVHD recipients is driven by alloimmune responses. Lethally irradiated C57BL/6 recipients were given whole spleen (10×10^{6}) plus TCD-BM (2.5×10^{6}) from MHC-matched but minor mismatched LP/J donors **(A)** or from syngeneic C57BL/6 donors **(B).** 21 days after HCT, splenocytes were stained for CD4, CD44, PSGL-1, and CD62L. Gated CD4^{+}CD44^{hi} are shown as PSGL-1 versus CD62L. PSGL-1 low and CD62L low cells were gated as extrafollicular CD4^{+} T cells. Percentages of PSGL-1^{lo}CD62L^{lo} cells among CD4^{+}CD44^{hi} cells are shown as mean ± SE (n=6). *** $p<0.001$, NS unpaired 2-tailed student t test.

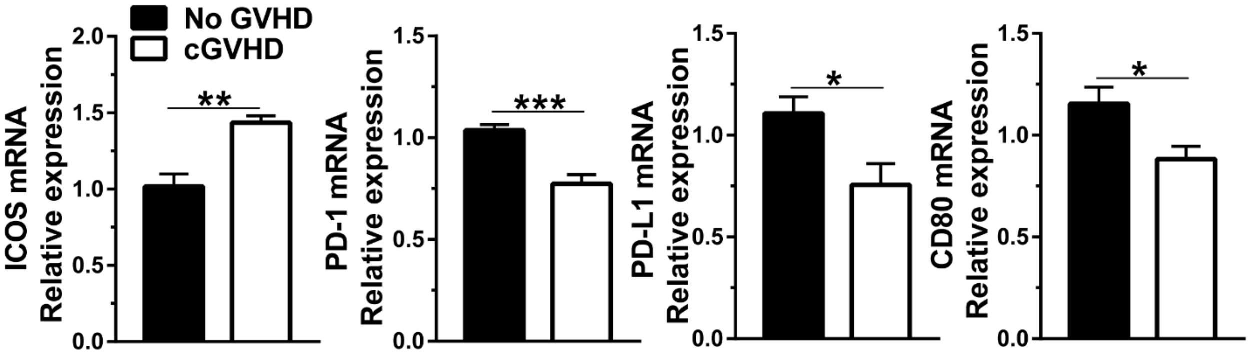

Supplementary Fig. 15 mRNA levels of ICOS, PD-1, PD-L1 and CD80 tested by real time PCR. 21 days after HCT, splenocytes from no GVHD or chronic GVHD recipients given wild-type C57BL/6 donors were harvested and stained for CD4, CD44, PSGL-1 and CD62L. $CD44^{hi}CD62L^{lo}PSGL\text{-}1^{lo}CD4^{+}$ T cells were sorted and used for RNA isolation. mRNA level of was measured by real time PCR. Mean ± SE is shown of 4 replicate experiments. Each experiment is combined from 8 mice. *p<0.05, **p<0.01, *** p<0.001 unpaired 2-tailed student t test**.**

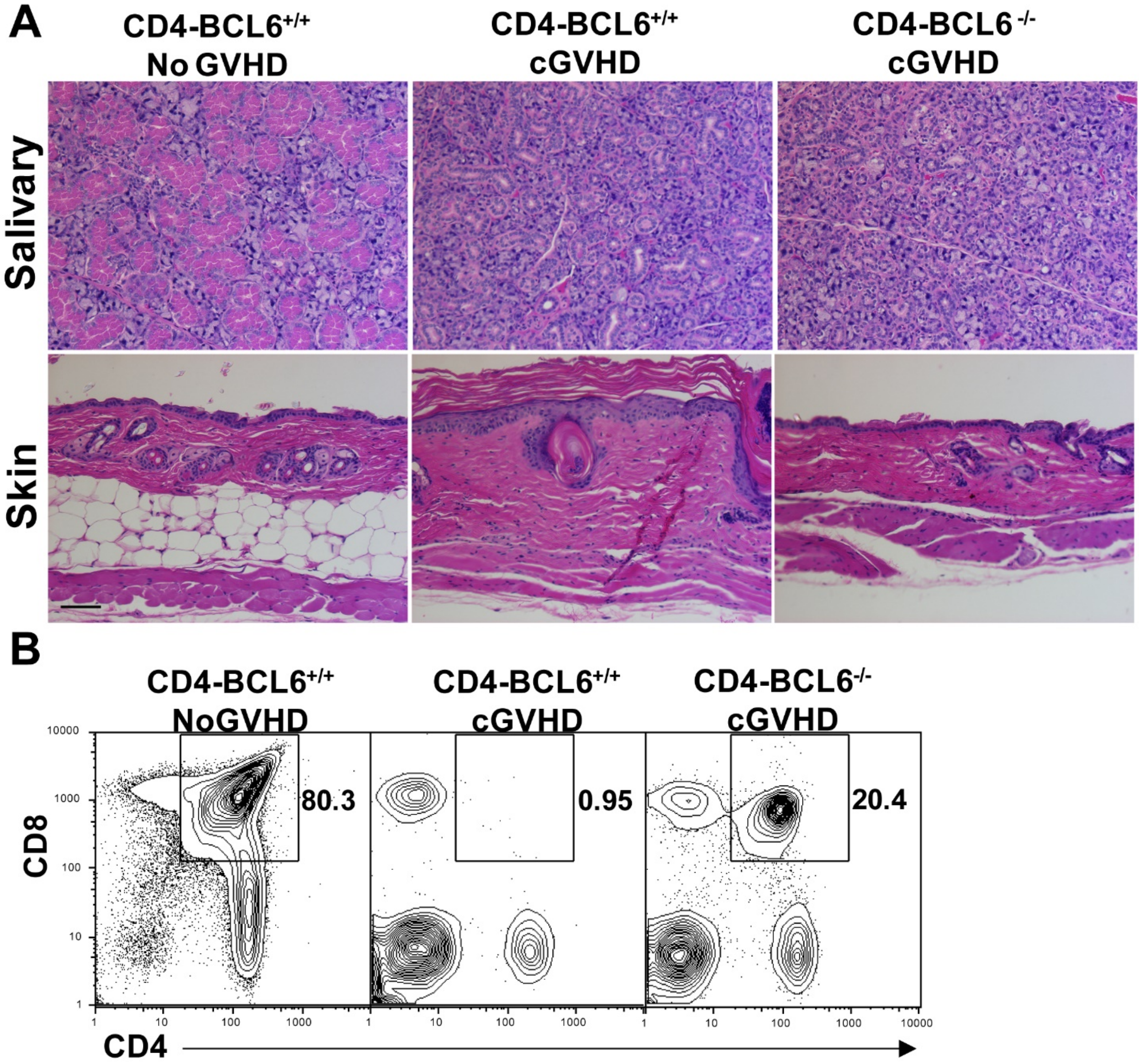

Supplementary Fig. 16 Little skin damage and reduced thymus damage in recipients given CD4-BCL6$^{-/-}$ grafts. BALB/c recipients were irradiated (850 cGy) and given 2.5x10^{6} TCD-BM alone or 2.5x10^{6} TCD-BM plus 1X10^{6} splenocytes from either WT or CD4-BCL6$^{-/-}$ C57BL/6 donors. (A) 60 days after HCT, salivary gland and skin specimens were harvested and used for H&E staining. One representative of 6 recipients in each group is shown. Scale bar, 50μm. (B) 60 days after transplantation, thymus specimens were harvested and stained for CD4 and CD8. Gated mononuclear cells are shown as CD4 versus CD8. One representative of 6 recipients in each group is shown.

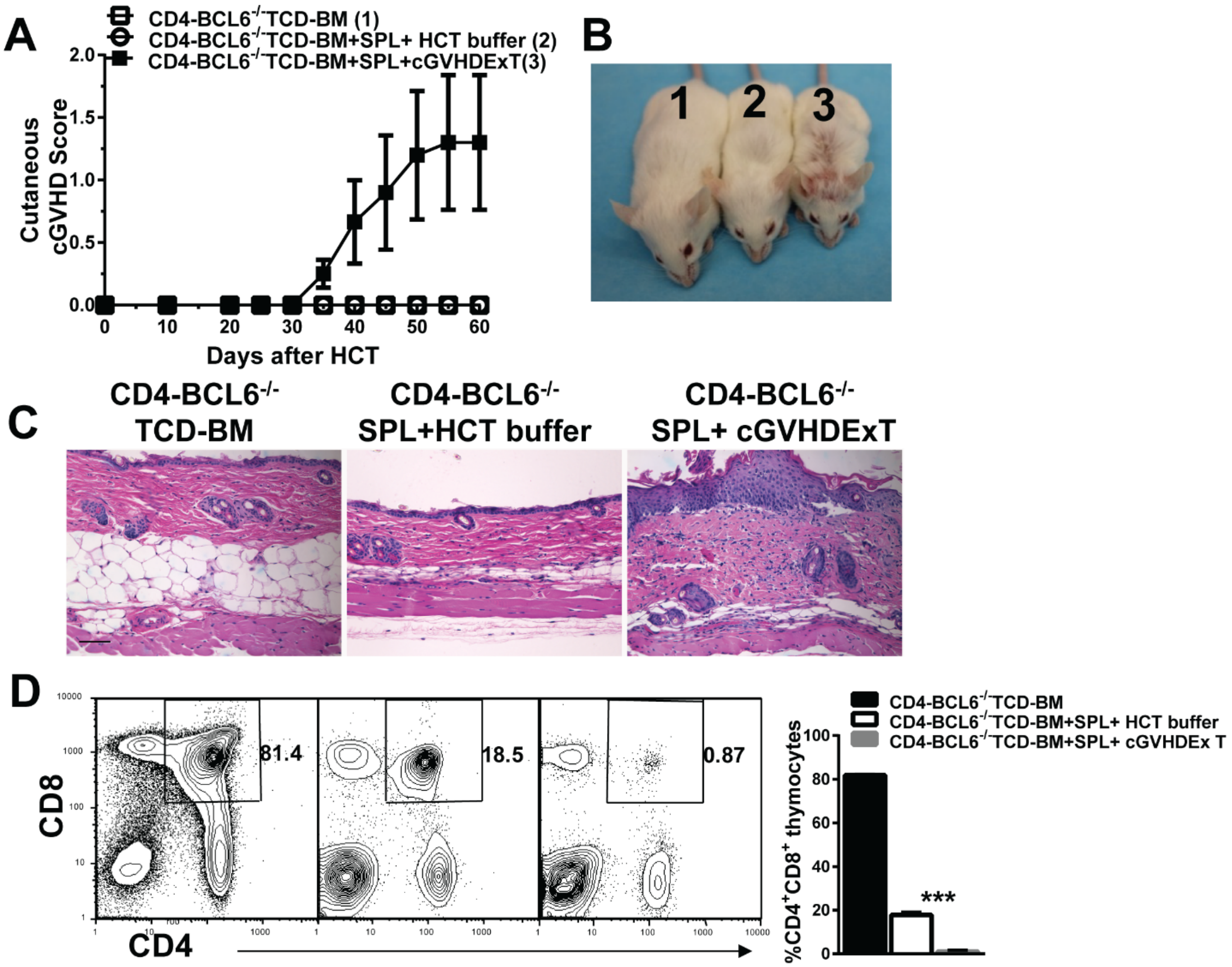

Supplementary Fig. 17: Adoptive transfer of extrafollicular PSGL-1^{lo}CD4^{+} T cells augments cutaneous chronic GVHD. BALB/c recipients were conditioned with 850 cGy TBI and given TCD-BM (2.5x10^{6}) alone or TCD-BM plus splenocytes (1X10^{6}) from either B-BCL6$^{-/-}$ or CD4-BCL-6$^{-/-}$ C57BL/6 donors. 21 days after transplantation, sorted PSGL-1^{lo}CD4^{+} T cells from GVHD recipient given B-BCL6$^{-/-}$ transplants were injected into recipients given CD4-BCL6$^{-/-}$ transplants. Recipients given HCT buffer were used as control. Recipients were monitored for chronic GVHD development for up to 60 days. **(A)** Cutaneous cGVHD scores (Group 3 vs Group 2: P<0.001 two-way ANOVA). There were 8 mice per group combined from two replicate experiments. **(B)** Picture taken at D60 after HCT, one representative is shown of 8.1-no GVHD,2-HCT buffer,3-cGVHDExT **(C)**H&E staining of skin, one representative is shown of 6. Scale bar, 50µm. **(D)** 60 days after first transplantation, thymus specimens were harvested and stained for CD4 and CD8. Gated mononuclear cells are shown as CD4 versus CD8 (n=6) *** p<0.001 unpaired 2-tailed student t test.

.

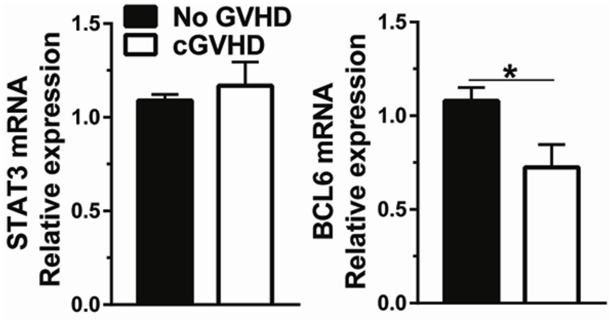

Supplementary Fig 18 mRNA levels of STAT3 and BCL6 tested by real time PCR. 21 days after HCT, splenocytes from no GVHD or chronic GVHD recipients given wild-type C57BL/6 donors were harvested and stained for CD4, CD44, PSGL-1 and CD62L. $CD44^{hi}CD62L^{lo}PSGL\text{-}1^{lo}CD4^{+}$ T cells were sorted and used for RNA isolation. mRNA level of Stat3 and BCL6 was measured by real time PCR. Mean ± SE is shown of 4 replicate experiments. Each experiment is combined from 8 mice. *p<0.05, unpaired 2-tailed student t test**.**

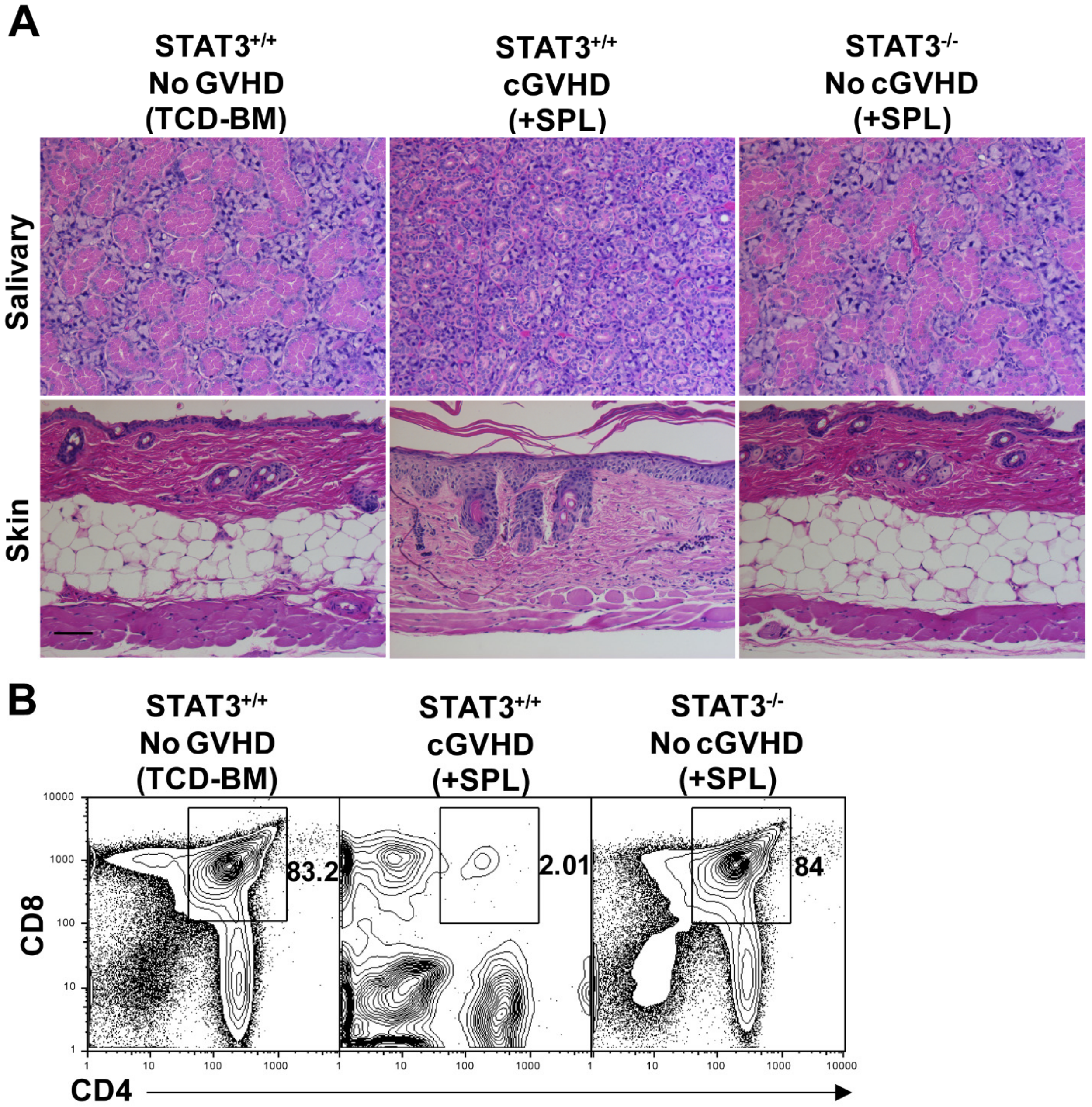

Supplementary Fig. 19: No damage in thymus, skin and salivary gland in recipients given CD4-STAT3$^{-/-}$ grafts. BALB/c recipients were irradiated (850 cGy) and given 2.5x10^{6} TCD-BM alone or 2.5x10^{6} TCD-BM plus 1X10^{6} splenocytes from either WT or CD4-STAT3$^{-/-}$ C57BL/6 donors. (A) 60 days after HCT, salivary gland and skin specimens were harvested and stained with H&E. One representative of 6 recipients in each group is shown. Scale bar, 50μm (B) 60 days after transplantation, thymus specimens were harvested and stained for CD4 and CD8. Gated mononuclear cells are shown as CD4 versus CD8. One representative of 6 recipients in each group is shown.

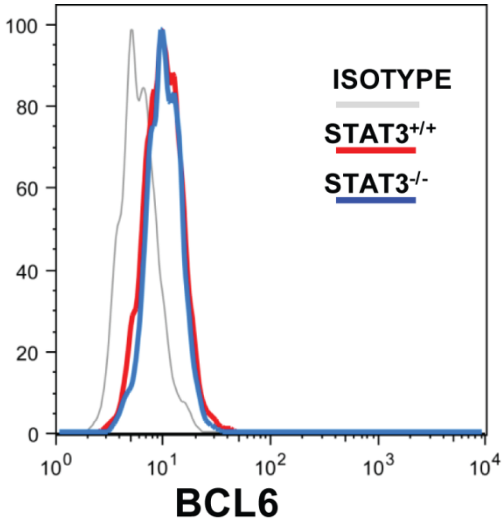

Supplementary Fig.20 The STAT3 deficient PSGL-1^{lo}CD4^{+} T expressed similar levels of BCL6 to wild type control. Twenty-one days after HCT, spleen were harvested from recipients given 2.5x10^{6} TCD-BM plus 1X10^{6} splenocytes from WT or CD4-STAT3$^{-/-}$ C57BL/6 donors. Splenocytes were stained with CD4, CD44, PSGL-1 and CD62L. BCL6 expression on PSGL-1^{lo}CD4^{+} were shown. One representative of 6 recipients is shown.

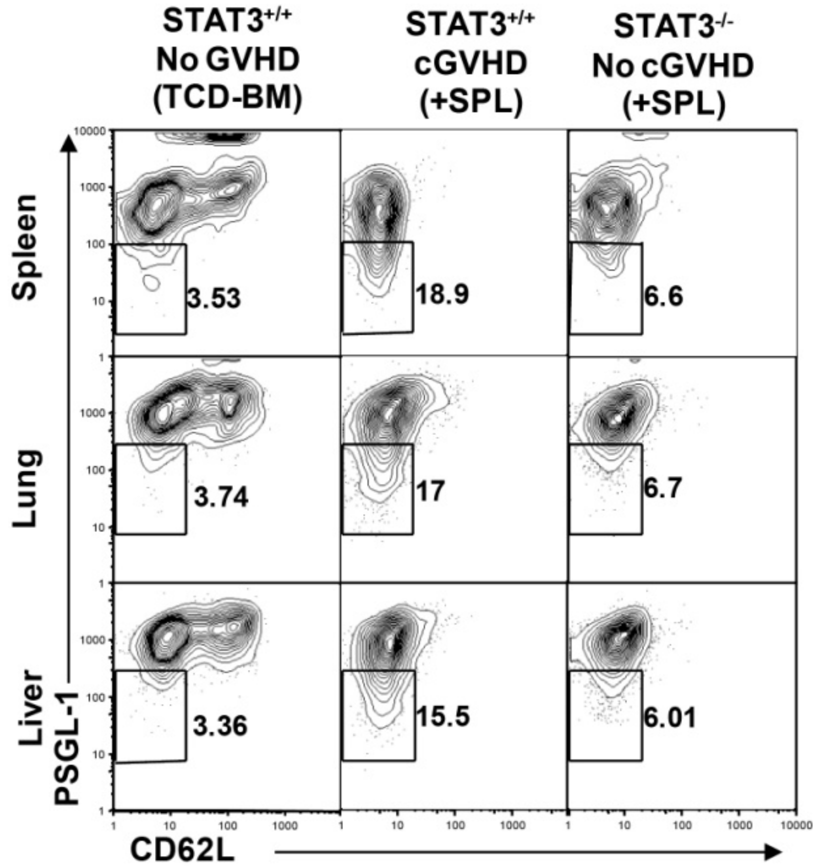

Supplementary Fig. 21: Reduced PSGL-1^{lo}CD4^{+} T cells percentage in the spleen, lung, and liver of recipients given CD4-STAT3$^{-/-}$ grafts. Twenty-one days after HCT, spleen, lung and liver specimens were harvested from recipients given 2.5x10^{6} TCD-BM alone or plus 1X10^{6} splenocytes from WT or CD4-STAT3$^{-/-}$ C57BL/6 donors. Splenocytes and mononuclear cells isolated from lung and liver were stained with CD4, CD44, PSGL-1 and CD62L. Gated CD4^{+}CD44^{hi} are shown as PSGL-1 versus CD62L.

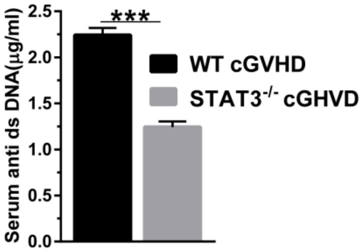

Supplementary Fig. 22 Serum anti-dsDNA levels of BALB/c recipients given wild-type or CD4-STAT3$^{-/-}$ grafts. BALB/c recipients were irradiated (850 cGy) and given 2.5x10^{6} TCD-BM plus 1X10^{6} splenocytes from wild-type, CD4-STAT3$^{-/-}$ C57BL/6 donors. Serum anti-ds DNA levels from recipients 45 days after transplantation are shown as mean ± SE (n=8). *** p<0.001 unpaired 2-tailed student t test.

.

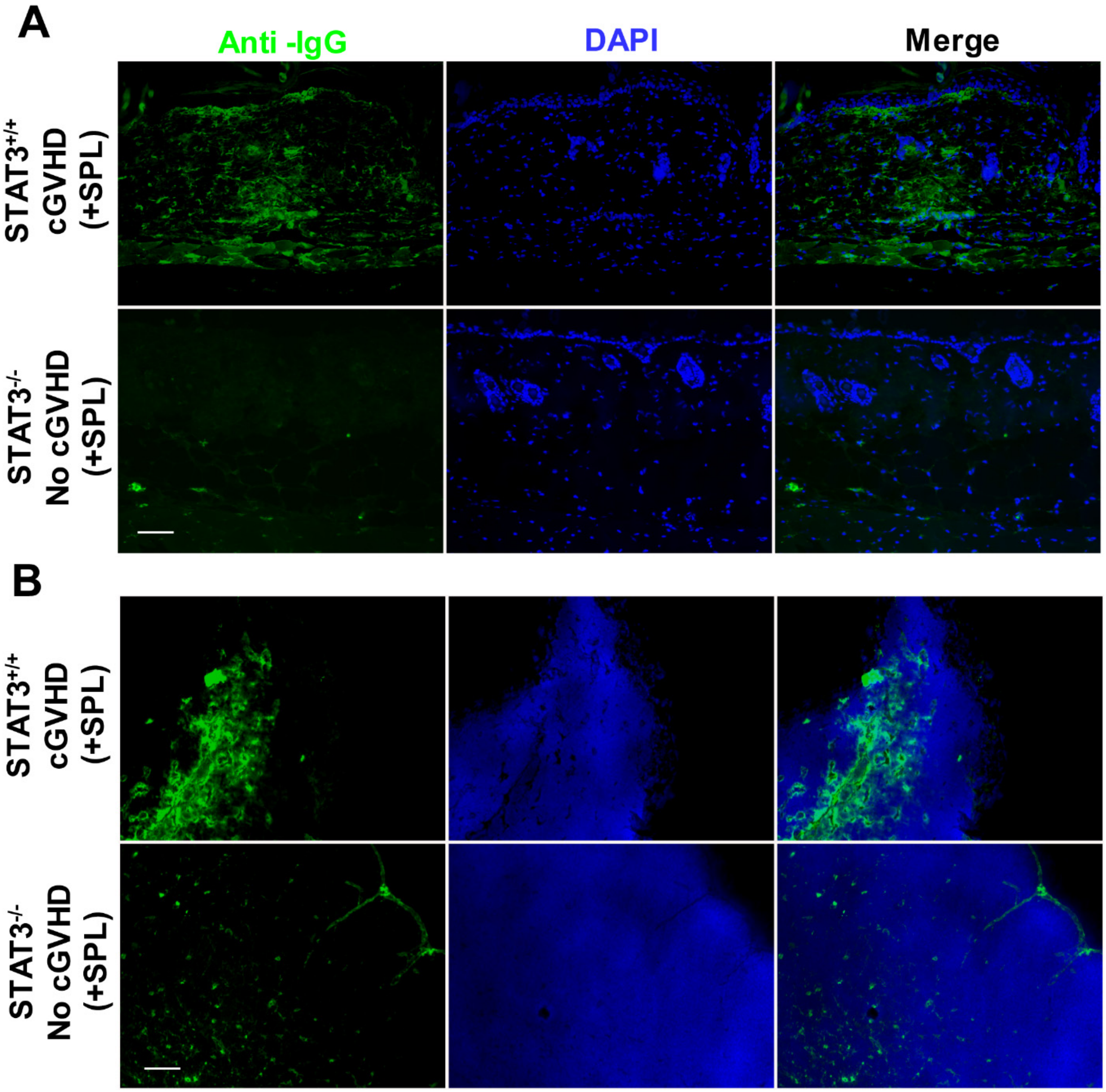

Supplementary Fig. 23 No obvious IgG deposition in the skin and thymus of recipients given CD4-STAT3$^{-/-}$ graft. BALB/c recipients were conditioned with 850 cGy TBI and transplanted with TCD-BM ($2.5x10^6$) and spleen cells ($1x10^6$) from WT or CD4-Stat3$^{-/-}$ C57BL/6 donors. 45 days after HCT, skin (A) and thymus (B) tissues were stained for tissue IgG deposition with rat-anti-mouse IgG-FITC. One representative is shown of 6 recipinets in each group. Scale bar, 50μm.

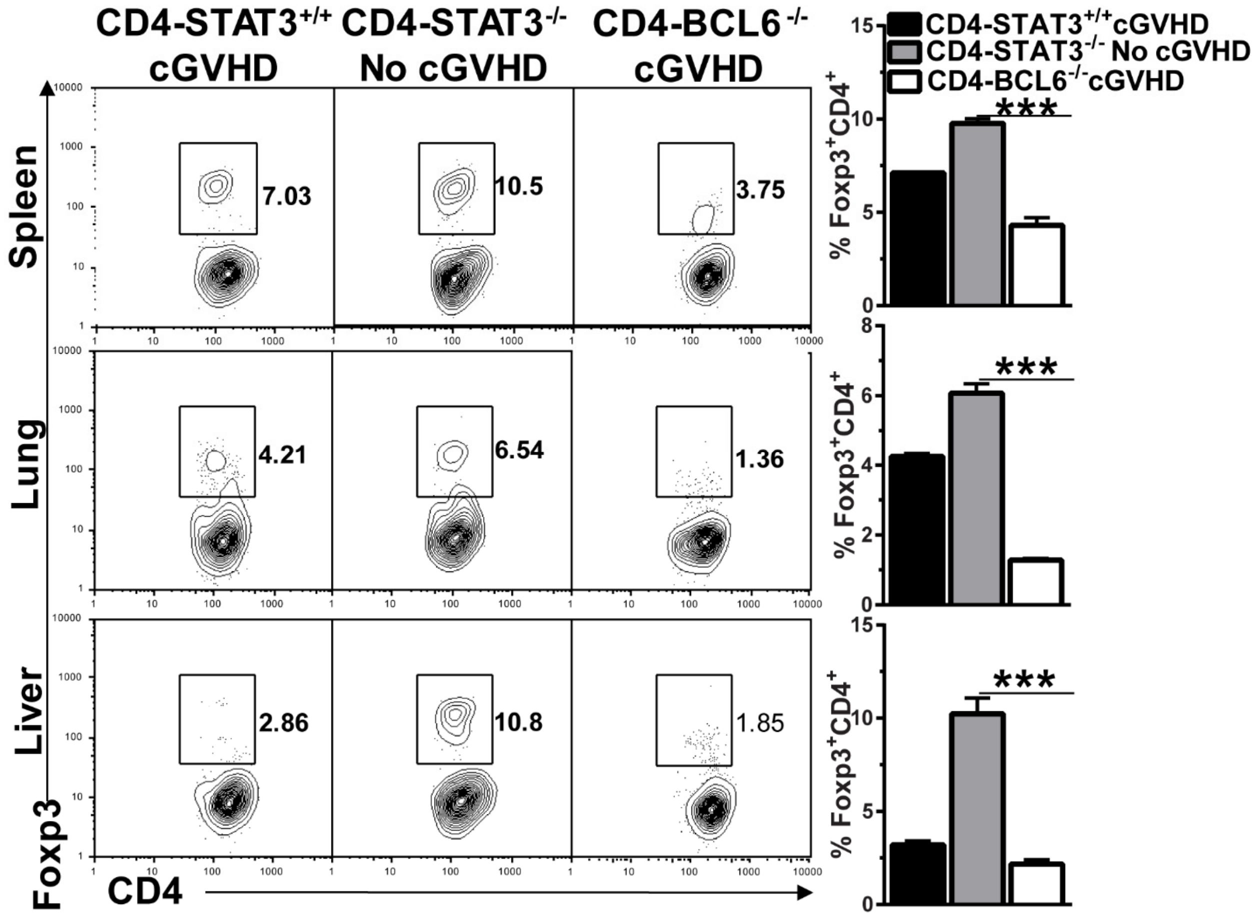

Supplementary Fig. 24 Treg in the spleen of recipients given grafts with Stat3$^{+/+}$, Stat3$^{-/-}$ or BCL6$^{-/-}$ CD4^{+} T cells. BALB/c recipients were irradiated (850 cGy) and given 2.5x10^{6} TCD-BM alone or 2.5x10^{6} TCD-BM plus 1X10^{6} splenocytes from either WT, CD4-STAT3$^{-/-}$ or CD4-BCL6$^{-/-}$ C57BL/6 donors. 45 days after transplantation, mononuclear cells from spleen, lung and liver were stained for CD4 and Foxp3. (A) Flow cytometry patterns are shown as CD4 versus Foxp3. Percentages of CD4^{+}Foxp3^{+}cells among CD4^{+} are shown as mean ± SE (n=6). *** $p<0.001$ unpaired 2-tailed student t test.

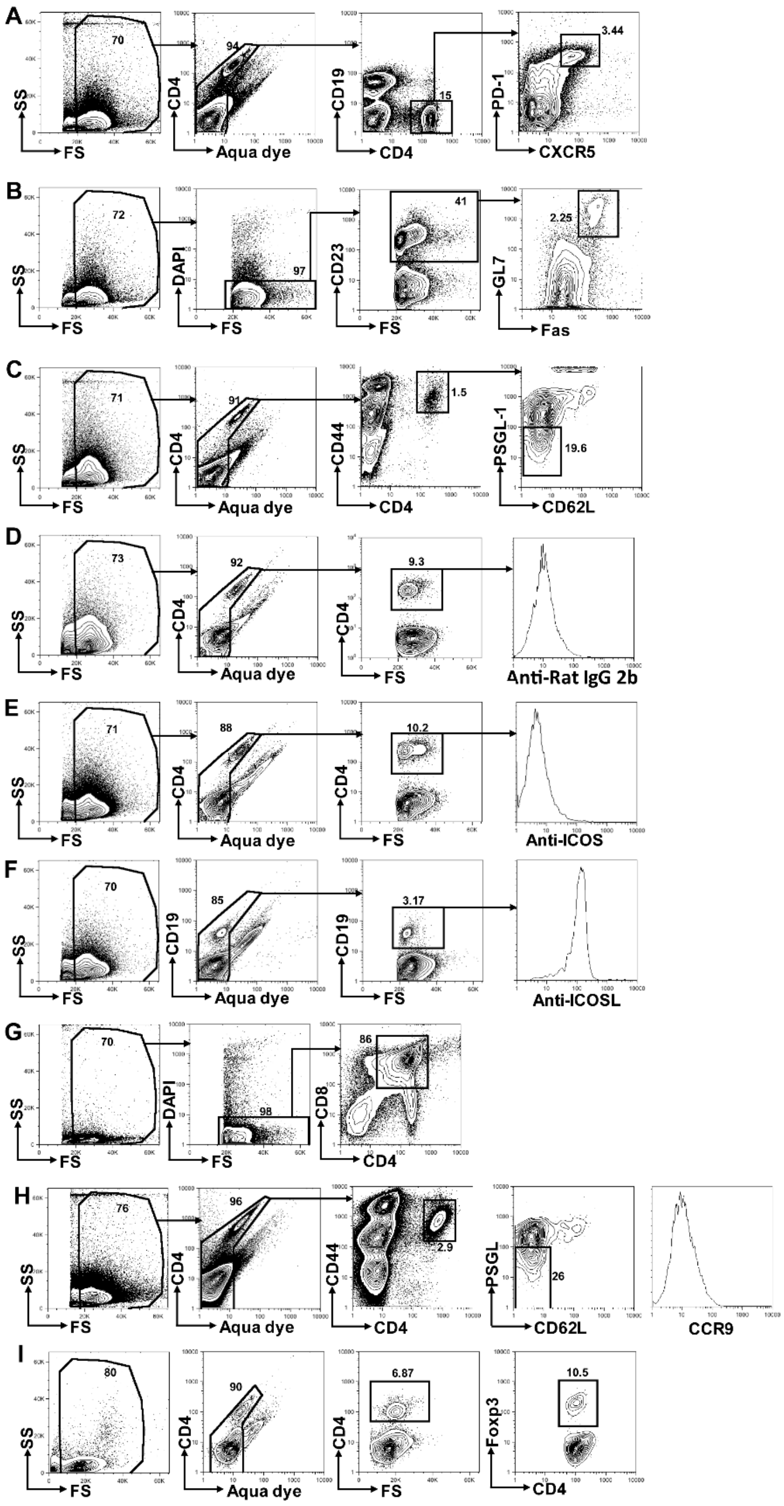

Supplementary Fig. 25 Gating strategies for flow cytometry analysis. Representative gating strategies to analyzes: (A) Percentage of Tfh shown in Fig. 2f, Supplementary Fig. 4 f and Supplementary Fig. 13. (B) Percentage of germinal centre B shown in Fig. 2g, Supplementary Fig. 4 G. (C) Percentage of PSGL-1^{lo}CD4^{+} T shown in Fig. 3 a&b, Fig. 5a, Fig. 6f, Fig. 8c, Supplementary Fig. 11, 14 & 21 and sorting. (D) Binding of Anti-Rat IgG2b shown in Fig. 5c. (E) Binding of Anti-ICOS shown in Fig. 5d. (F) ICOSL expression level on B cells shown in Fig. 5e. (G) CD4^{+}CD8^{+} thymocyte shown in Fig. 8 a&b, Supplementary Fig.1,16,17&19. (H) CCR9 expression level shown in Fig. 8d. Similar gating strategy to FasL expression level shown in Fig.8d. (I) Percentage of Treg shown in Supplementary Fig. 24.
